# Women’s attitudes and beliefs towards specific contraceptive methods in Bangladesh and Kenya

**DOI:** 10.1186/s12978-018-0514-7

**Published:** 2018-05-08

**Authors:** Kazuyo Machiyama, Fauzia Akhter Huda, Faisal Ahmmed, George Odwe, Francis Obare, Joyce N. Mumah, Marylene Wamukoya, John B. Casterline, John Cleland

**Affiliations:** 1 Faculty of Epidemiology and Population Health, London School of Hygiene and Tropical Medicine, Keppel Street, London, WC1E 7HT UK; 2 icddr,b, Dhaka, Bangladesh; 3 Population Council, Nairobi, Kenya; 4 African Population and Health Research Center, Nairobi, Kenya; 5 Institute for Population Research, Ohio State University, Columbus, USA

**Keywords:** Contraception, Contraceptive methods, Attitude, Belief, Satisfaction, Kenya, Bangladesh

## Abstract

**Background:**

Missing from the huge literature on women’s attitudes and beliefs concerning specific contraceptive methods is any detailed quantitative documentation for all major methods in low- and middle-income countries. The objectives are to provide such a documentation for women living in Matlab (rural Bangladesh), Nairobi slums and Homa Bay (rural Kenya) and to compare the opinions and beliefs of current, past and never users towards the three most commonly used methods (oral contraceptives, injectables and implants).

**Methods:**

In each site, 2424 to 2812 married women aged 15–39 years were interviewed on reproduction, fertility preferences, contraceptive knowledge and use, attitudes and beliefs towards family planning in general and specific methods. We analysed the data from round one of the prospective cohort study.

**Results:**

While current users typically expressed satisfaction and held more positive beliefs about their method than past or never users, nevertheless appreciable minorities of current users thought the method might pose serious damage to health, might impair fertility and was unsafe for prolonged use without taking a break. Larger proportions, typically between 25% and 50%, associated their method with unpleasant side effects. Past users of pills and injectables outnumbered current users and their beliefs were similar to those of never users. In all three sites, about half of past injectable users reported satisfaction with the method and the satisfaction of past implant users was lower.

**Conclusions:**

High levels of contraceptive use can clearly co-exist with widespread misgivings about methods, even those that are widely used. Serious concerns about damage to health, long term fertility impairment, and dangers of prolonged use without taking a break were particularly common in the Kenyan sites and these beliefs may explain the high levels of discontinuation observed in Kenya and elsewhere in Africa. This documentation of beliefs provides useful guidance for counselling and informational campaigns. The generally negative views of past users imply that programmes may need not only to improve individual counselling but also strengthen community information campaign to change the overall climate of opinion which may have been influenced by dissatisfaction among past users.

**Electronic supplementary material:**

The online version of this article (10.1186/s12978-018-0514-7) contains supplementary material, which is available to authorized users.

## Plain english summary

In low- and middle-income countries, women’s beliefs about specific contraceptive methods are not well understood. This study documents the beliefs about eight contraceptive methods among women living in Matlab (rural Bangladesh), Nairobi slums and Homa-Bay (rural Kenya) and compares the opinions of current, past and never users of the most commonly used methods (oral contraceptives, injectables and implants). In each site, we interviewed 2424 to 2812 married women aged 15–39 years. As expected, we found that current contraceptive users were typically satisfied and had more positive beliefs about their method than past or never users. Nevertheless, large minorities of current users thought that their method might cause serious health problems, impair future childbearing and was unsafe to use for a long time; higher proportions (25–50%) reported that their method use caused unpleasant side effects. Past users of pills and injectables outnumbered current users and their beliefs were similar to never users. In all three sites, about half of past injectable users reported satisfaction with the method but the satisfaction of past implant users was lower. Despite high contraceptive use in these populations, adverse and inaccurate beliefs about the major methods persist, particularly in Kenya. This study provides useful guidance for counselling and informational campaigns. The generally negative views of past users imply that programmes may need not only to improve individual counselling but also strengthen community information campaign to change the overall climate of opinion which may have been influenced by dissatisfaction among past users.

## Background

Over the past half century, an extensive literature has been accumulated on women’s attitudes and beliefs concerning contraception in general and specific methods. The extensive evidence for low- and middle-income countries (LMICs) falls into four main categories. The most common category comprises studies that examine attitudes towards and beliefs about contraception in general, with little or no distinction between specific methods [1–6]. The emphasis in many papers is on negative perceptions, often labelled as myths and misinformation. Commonly reported themes include the belief that use of modern methods will cause long term infertility, serious health damage, such as cancer and foetal abnormalities. In some studies, respondents associated contraception with promiscuity [2].

The second category concerns specific methods. Owing to the HIV pandemic, a huge literature on beliefs about condoms has been generated, much of which has been summarised by Maticka-Tyndale [7]. A review of perspectives on intra-uterine devices (IUDs) identified 14 studies from Africa, Asia and Latin America [8]. Positive features of this method included its high effectiveness and long-acting nature while commonly expressed concerns were health risks including cancer, ectopic pregnancy, infertility and harm to the husband during intercourse. A study in Kenya found that postpartum women preferred implants over IUDs because of less pain, less infringement of modesty and preference for a superficial insertion in the upper arm than in the uterus [9]. The literature on hormonal methods shows the dominant concerns to be side effects such as nausea, dizziness and weight change, menstrual disruption and infertility [10–12]. In both Mali and Kenya, many women believed that oral contraceptives accumulate inside the body, causing infertility or a variety of diseases [13, 14].

The most influential, though indirect, body of evidence on views about contraceptive methods is based on self-reported reasons for non-use or discontinued use of contraception. The dominant source of data is the Demographic and Health Surveys (DHSs). The most recent analysis of reasons for non-use among married women not wishing to get pregnant found that, in most of the 52 countries studied, 20–33% cited side effects or health concerns. In 21 countries, this category of reason was the most common [15]. Moreover, in many countries, the majority of women giving this reason for contraceptive avoidance had previously used a modern method. Thus fear of side effects (and perhaps concerns about health) is partly based on personal experience and partly on hearsay evidence from friends or from media [16].

Reasons for discontinued use is more useful than reasons for non-use in distinguishing method-specific concerns. For oral contraceptives, injectables and IUDs, side effects or health concerns are the most common reason for stopping use. For condoms, objections from the husband or desire for a more effective method are the key reasons while for withdrawal and periodic abstinence accidental pregnancy dominates [17]. One limitation of this type of evidence is lack of comparable data for continuing users who perhaps are equally concerned about side effects or health risks as discontinued users but persist with use because of stronger motivation to avoid pregnancy or for some other reason.

The final main type of studies addresses the characteristics of contraceptive technology that women judge to be important. The single largest study of this type involved focus group discussions with 576 women in seven countries, six of which were LMICs [18]. Widespread agreement was found on the key importance of effectiveness and reversibility. Opinions varied by site on the desirability of a quick return to fertility after stopping the method and of the possibility of clandestine use. Most women found amenorrhea as a result of hormonal method use to be disturbing and similar results were found in Ghana and Nigeria [19, 20]. In a systematic review of contraceptive attributes that women take into account when choosing a method drawing on studies in USA and Europe as well as LMICs, ease of use, frequency of use, return to fertility, and side effects and health concerns were the most commonly mentioned [21].

Conspicuously and surprisingly lacking from the evidence-base for LMICs is detailed, quantitative documentation of the beliefs of women concerning all major methods of contraception. Our study addresses the omission by presenting descriptive survey data on method-specific beliefs in three populations. The data presented here form part of a wider prospective cohort study whose aims are to advance the understanding of reasons for unmet need for contraception and measurement of fertility preferences. The detailed rationale and study protocol may be found elsewhere [22]. In brief, its conceptual underpinnings posit that adverse method-specific beliefs constitute one cause of unmet need and unintended pregnancy, alongside four other factors (generic hostility to contraception, partner-related factors, weak or inconsistent fertility preferences and low perceived risk of pregnancy). The prospective design of the study will permit assessment of the relative power of these factors to predict method-specific adoption, continuation and pregnancy-incidence. Here, however, we present data from round one with the aim of documenting method-specific beliefs and of comparing the beliefs concerning oral contraceptives, injectables and implants of current, past and never users.

## Methods

The survey was conducted in the icddr,b service area and the government service area of the Matlab Health and Demographic Surveillance System (HDSS), Bangladesh, the Nairobi Urban Health and Demographic Surveillance System (NUHDSS) in two slums in Nairobi, Kenya, and Homa Bay County in rural Western Kenya. Details of the three study population have been published [22], but, in summary, contraception has been well established in Matlab for decades and the total fertility rate (TFR) is 2.7 [23]. The population is predominantly Muslim. In Nairobi slums, the TFR is 3.5 and HIV prevalence is 8% in the adult population [24, 25]. In the two slums covered by the NUHDSS, the population is ethnically diverse, the majority belong to a Christian faith and mobility is high. Homa Bay’s population is predominantly rural, Christian and of Luo ethnicity. It has a high TFR of 5.2, high unmet need for contraception and a severe HIV epidemic with an infection level in the adult population of 26% [26]. All married or co-habiting women aged between 15 and 39 years residing in the three sites were eligible for the study. We set a target sample size for each site of 2600. The sample size was determined to detect a 30% difference in reproductive outcomes (pregnancy, use and non-use of contraceptives) at 95% confidence level and 80% power with an assumption of 10% non-response rate [27].

Data collection was carried out between August and December 2016. Respondents were randomly sampled from eligible female residents from the HDSS databases in the Matlab and Nairobi sites. In Homa Bay two-stage cluster sampling was used. A total of 34,308 women were identified as being married and aged between 15 and 39 years in the Matlab HDSS database. Out of these, 3109 were randomly selected and 2605 women completed the interviews. The Nairobi HDSS database identified a total of 5905 married or cohabiting women who were eligible for inclusion. Out of these, 3093 women were randomly sampled and interviews were completed with 2812 of the eligible women.

In Homa-Bay, we randomly selected 12 sub-locations (the smallest administrative unit in Kenya) in each three purposely identified sub-counties in Homa Bay County. All households with currently married or co-habiting women aged 15–49 years in the selected sub-locations were identified with the help of the local administration. Subsequently, the sampling frame was generated by listing all individuals in these households. In the second stage, 3118 were randomly sampled out of 5424 eligible women. A total of 2424 women completed the interviews.

All respondents provided written consents after having been informed about the objectives, procedures, benefits and risks of the study using an information sheet and informed consent form. All married and co-habiting adolescents aged 15–17 years were considered emancipated minors for whom parental permission is not required.

The questionnaire was developed through literature review and consultation with experts. We reviewed existing literature and more than 30 questionnaires on fertility preferences and reasons for non-use of family planning fielded in high-, middle- and low-income countries including instruments from the DHS, the Determinants of Unintended Pregnancy Risk study in New Orleans; the US- based National Survey of Family Growth (NSFG); and the Fog Zone study by the Guttmacher Institute. A questionnaire was developed and reviewed through consultation with dozens of experts in the field. The questionnaire was further refined after pre-testing.

Structured interviews, lasting on average for 45–60 min, were conducted using the local languages following a 7 to 10-day training. Very similar questionnaires were used in all three sites covering the following topics: background, reproduction, contraceptive knowledge and use, beliefs and attitudes towards contraception in general and specific methods and fertility preferences of the women. Two summary measures of method-specific beliefs were generated. A net positive score is the mean percent difference between positive and negative responses across nine women’s attributes, ignoring “don’t know” and perceived husband’s approval. Positive responses refer to perceptions that a method is easy to obtain and to use, very effective in preventing pregnancy and safe to use for a long time without a break, does not cause health problems, unpleasant side effects, menstrual disruption or infertility, and that half or more of friends, relatives, neighbours (i.e. social network) had used the method and their experiences were satisfactory. A familiarity measure which is simply the mean percent responding “don’t know” across eight attributes excluding experiences of method use among women’s social network and perceived husband’s approval. In addition, past and current users of a method were asked whether they were satisfied or dissatisfied with use.

Descriptive analysis and chi-square tests were carried out to assess differences in beliefs and attitudes among current, past and never users of pills, injectables and implants. Clustering was taken into account for the statistical analyses when analysing the data from Homa-Bay. We used STATA SE version 15.0 for the data analysis.

Ethical approvals for this study were obtained from the Institutional Review Boards of the countries and three participating institutions.

## Results

The number of women successfully interviewed was 2424 in Homa Bay, 2605 in Matlab, and 2812 in Nairobi (Additional file 1: Table S1). The average ages of women in the three sites were closely similar, 28 or 29 years (Table 1). About 70% in Matlab had secondary or higher schooling, compared with 40% in Nairobi and 23% in Homa Bay. The mean number of surviving children was lowest in Matlab, highest in Homa Bay and intermediate in Nairobi. Women in Matlab were much more likely to express a desire to stop childbearing than those in the Kenyan sites. In Nairobi, 75% were currently using a method of contraception, with injectables followed by implants, as the dominant methods. In Homa Bay, the level of current use was 65%, with a method-mix similar to that in Nairobi. Current use was lowest in Matlab (59%), where oral contraception pills and injectables were the favoured methods. But this unexpected result, in view of the far lower fertility rate in Matlab, may largely reflect the fact that 34% of husbands had been away for three months or longer preceding the date of interview, mainly as migrant workers, thus reducing the need for pregnancy-protection.

**Table 1:** Selected Background of Respondents

Characteristics	Matlab	Nairobi	Homa Bay
Educational attainment (%)			
Primary or less	30.4	60.4	77.5
Secondary or higher	69.6	39.6	22.5
Current FP method use (%) (1)			
No use	40.7	24.6	35.5
Pills	25.4	8.4	2.7
Injectables	17.7	32.4	26.8
Implants	2.2	19.8	17.9
Condoms	5.5	1.7	8.1
Other	8.5	13.2	8.9
Women want no more children (%)	44.8	27.0	29.2
Mean age of women	29.3	29.1	27.9
Mean number of living children	1.9	2.3	3.4
Total Number of Respondents	2605	2812	2424

(1) If more than one method is used, only the most effective method is included in this table

### Generic attitudes

Though the focus of this paper is on method-specific beliefs, we start by presenting information on attitudes towards contraceptive use in general. In all three sites, over 90% of women reported that they supported family planning, but 25% in Homa Bay and 12% in Nairobi perceived their husbands to be opposed (Additional file 1: Table S2). Use of contraception by women’s social network of friends, neighbours, and relatives was thought to be common and their attitudes were favourable in all three sites. Conversely, only around half in Matlab (51%) and Homa Bay (47%) and three-fourths (76%) in Nairobi perceived that their religion supports family planning.

Large majorities considered the following features of methods to be very important: effectiveness, no health risk, lack of unpleasant side effects, no effect on menstruation, and ease of access and use (Additional file 1: Table S3). The amenability of a method for clandestine use and use for a long time without the need for re-supply were endorsed as very important by smaller majorities.

### Method-specific beliefs and opinions: Matlab

A total of ten opinions and beliefs was ascertained for all women who have ever heard of each of six modern methods (Tables 2, 3, 4, 5, 6 and 7) and two traditional methods (Additional file 1: Table S4). Because of the wide array of information, the discussion here will be highly selective.

**Table 2:** Percentage of women with specific opinions, amongst those who have heard of methods (Matlab)

Attributes	Pills	Injectables	Implants	IUD	Condoms	Female sterilisation
Access						
Easy	97.2	90.4	66.1	69.5	87.1	71.5
Hard	2.2	8.0	24.9	24.1	9.6	23.9
Don’t know/unsure	0.6	1.6	9.0	6.4	3.3	4.6
Effectiveness						
Yes	99.3	98.6	97.0	97.3	88.0	97.6
No	0.5	1.0	0.5	0.5	8.3	0.8
Don’t know	0.2	0.4	2.5	2.2	3.7	1.6
Cause health problems						
Yes, serious	3.0	7.3	8.3	10.3	3.5	6.7
Yes, not serious	20.5	33.0	15.3	15.1	3.0	8.6
No	69.1	40.2	11.1	8.0	58.6	27.3
Don’t know	7.4	19.5	65.3	66.6	34.9	57.4
Interfere with menstruation						
Yes	7.6	56.8	15.7	16.5	–	4.3
No	84.4	21.6	12.9	12.0	–	33.5
Don’t know	8.0	21.6	71.4	71.5	–	62.3
Cause unpleasant side effect						
Yes	26.2	37.8	18.7	21.5	7.9	7.7
No	66.1	41.7	12.2	9.0	54.1	30.8
Don’t know	7.7	20.5	69.1	69.5	38.0	61.5
Unsafe to use for a long time						
Yes, should take a break	21.2	23.4	20.4	21.2	23.4	–
No, safe for long time	74.3	67.1	52.7	53.7	53.7	–
Don’t know	4.5	9.5	26.9	25.1	22.9	–
Cause infertility						
Yes, perhaps	8.8	6.5	4.1	4.1	–	–
No	85.2	84.8	74.8	76.2	–	–
Don’t know	6.0	8.7	21.1	19.7	–	–
FP use among friend, relatives						
Most	25.0	17.0	0.7	0.9	1.2	0.8
About half	11.8	14.9	1.3	1.3	0.3	0.7
Few	54.2	52.2	41.2	36.1	31.1	52.3
None	4.2	8.2	28.1	31.8	14.5	21.1
Don’t know	4.8	7.7	28.6	29.9	52.9	25.1
Experiences of friend, relatives (1)						
Satisfactory	95.6	89.5	77.2	75.5	82.4	87.5
Unsatisfactory	2.2	5.6	8.6	10.2	3.2	2.0
Mixed/Don’t know	2.2	4.8	14.2	14.3	14.4	10.5
Husband’s approval						
Approve	89.4	71.2	19.9	22.0	37.5	14.2
Disapprove	7.5	22.6	64.2	64.4	52.7	70.8
Don’t know	3.1	6.2	15.9	13.6	9.8	15.0
Mean net positive score (2)	61.9	36.2	24.5	23.6	45.8	31.8
Mean % of don’t know (3)	4.9	11.2	36.7	36.4	26.0	35.4
TOTAL(N)	2600	2583	2253	1917	2572	2544

(1) Amongst those knowing some friends were using the methods

(2) Mean percent difference between percentages of women who gave positive and negative responses among the above attributes except husband’s approval

(3) Mean percent of women who said “don’t know” to the above attributes except experiences of a method in social network and husband’s approval

**Table 3:** Percentage of women with specific opinions by use status (Matlab)

Attributes	Pills			Injectables			Implants		
	Current users	Past users	Never users	Current users	Past users	Never users	Current users	Past users	Never users
Access									
Easy	98.6	98.3	92.4	98.5	95.5	84.0	96.5	87.5	64.7
Hard	1.4	1.5	5.0	1.5	4.2	13.0	1.8	12.5	25.8
Don’t know/unsure	0.0	0.2	2.6	0.0	0.3	3.0	1.7	0.0	9.5
Effectiveness									
Yes	99.8	99.4	98.5	98.9	99.1	98.1	100.0	96.4	96.9
No	0.2	0.6	0.6	1.1	0.8	1.1	0.0	3.6	0.5
Don’t know	0.0	0.0	0.9	0.0	0.1	0.8	0.0	0.0	2.6
Cause health problems									
Yes, serious	0.9	3.8	3.3	2.2	11.0	6.7	1.8	28.6	8.0
Yes, not serious	10.6	23.6	24.7	22.6	43.3	29.5	24.6	35.7	14.5
No	88.5	72.4	36.6	75.3	45.5	23.6	71.9	28.6	9.0
Don’t know	0.0	0.2	35.4	0.0	0.2	40.2	1.7	7.1	68.5
Interfere with menstruation									
Yes	6.0	9.0	5.8	67.9	72.2	41.8	43.9	53.6	14.0
No	93.8	90.6	56.6	31.0	27.5	14.0	52.6	39.3	11.1
Don’t know	0.2	0.4	37.6	1.1	0.3	44.2	3.5	7.1	74.9
Cause unpleasant side effect									
Yes	14.1	31.9	26.5	26.3	52.3	31.8	29.8	57.1	17.4
No	85.8	67.7	37.9	73.3	47.3	26.0	66.7	37.5	10.1
Don’t know	0.2	0.4	36.6	0.4	0.4	42.2	3.5	5.4	72.5
Unsafe to use for a long time									
Yes, should take a break	18.9	24.7	14.8	24.5	27.0	20.5	36.8	37.5	19.4
No, safe for long time	79.8	73.4	69.9	74.4	72.1	60.9	61.4	53.6	52.5
Don’t know	1.3	1.9	15.2	1.1	0.9	18.6	1.8	8.9	28.1
Cause infertility									
Yes, perhaps	6.5	10.3	7.6	6.1	7.1	6.2	3.5	0.0	4.3
No	90.9	85.9	76.6	91.8	89.9	78.6	87.7	91.1	74.0
Don’t know	2.6	3.8	15.8	2.1	3.0	15.2	8.8	8.9	21.7
FP use among friends, relatives									
Most	32.5	24.2	18.0	27.8	20.3	10.7	1.8	1.8	0.7
About half	15.0	10.5	11.3	18.0	14.7	13.8	7.0	5.3	1.1
Few	48.8	56.1	55.8	47.9	54.3	52.3	71.9	67.9	39.7
None	1.8	5.0	5.2	3.0	6.2	11.6	8.8	21.4	28.8
Don’t know	1.9	4.2	9.7	3.3	4.5	11.6	10.5	3.6	29.7
Experiences of friends, relatives (1)									
Satisfactory	98.4	95.3	92.4	95.8	88.8	87.3	93.5	71.4	76.6
Unsatisfactory	0.5	3.5	1.3	2.6	9.7	3.7	4.3	21.4	8.2
Mixed/Don’t know	1.1	1.3	6.3	1.6	1.5	9.0	2.2	7.1	15.1
Husband’s approval									
Approve	98.8	94.9	63.8	97.6	88.9	48.9	96.5	67.9	16.6
Disapprove	1.2	4.6	22.7	2.4	10.6	38.6	3.5	25.0	66.8
Don’t know	0.0	0.5	13.5	0.0	0.5	12.5	0.0	7.1	16.6
Satisfied with use									
Satisfied	92.2	69.7	–	91.3	51.1	–	87.7	39.3	–
Unsatisfied	4.8	27.7	–	8.0	47.2	–	10.5	51.8	–
Mixed/Neither	3.0	2.6	–	0.7	1.7	–	1.8	8.9	–
Mean net positive score (2)	74.8	60.9	48.8	53.3	34.7	31.0	45.8	19.2	24.0
Mean % of don’t know (3)	0.8	1.4	19.2	1.0	1.2	22.0	3.9	5.1	38.4
TOTAL(N)	662	1399	539	461	877	1245	57	56	2140

(1) Amongst those knowing some friends were using the methods

(2) Mean percent difference between percentages of women who gave positive and negative responses among the above attributes except husband’s approval

(3) Mean percent of women who said "don’t know" to the above attributes except experiences of a method in social network and husband’s approval

Note: Chi-square test was conducted to assess associations between each of the above attributes and use status. The *p*-values were all 0.01 or smaller except the perceptions on effectiveness of injectables (*p* = 0.067)

**Table 4:** Percentage of women with specific opinions, amongst those who have heard of methods (Nairobi)

Attributes	Pills	Injectables	Implants	IUD	Condoms	Female sterilisation
Access						
Easy	92.3	96.7	89.7	70.0	88.3	41.8
Hard	7.4	3.1	10.0	28.7	11.0	56.7
Don’t know/unsure	0.4	0.2	0.3	1.3	0.7	1.4
Effectiveness						
Yes	69.8	89.1	89.2	70.0	61.1	68.4
No	29.9	10.8	10.5	28.1	38.1	31.1
Don’t know	0.3	0.1	0.3	1.8	0.8	0.5
Cause health problems						
Yes, serious	12.5	18.5	23.2	35.4	4.0	30.0
Yes, not serious	28.8	37.1	36.2	24.6	11.6	13.5
No	58.2	44.2	40.1	38.1	83.4	54.2
Don’t know	0.6	0.2	0.5	1.9	1.1	2.3
Interfere with menstruation						
Yes	40.5	78.0	66.5	48.9	–	30.3
No	58.5	21.5	32.6	47.3	–	63.8
Don’t know	1.0	0.5	0.9	3.8	–	5.9
Cause unpleasant side effect						
Yes	47.2	59.9	58.7	58.7	18.0	40.6
No	52.4	40.0	41.0	39.7	81.2	56.5
Don’t know	0.5	0.1	0.3	1.5	0.8	2.9
Unsafe to use for a long time						
Yes, should take a break	70.1	62.9	56.9	65.2	48.8	–
No, safe for long time	29.5	36.7	42.7	33.3	49.6	–
Don’t know	0.4	0.4	0.4	1.5	1.6	–
Cause infertility						
Yes, perhaps	13.8	28.0	21.8	18.9	–	–
No	85.6	71.6	77.4	79.2	–	–
Don’t know	0.5	0.4	0.8	1.9	–	–
FP use among friends, relatives						
Most	20.2	63.5	38.2	5.7	11.4	1.7
About half	7.1	9.9	13.7	2.9	2.4	0.6
Few	52.2	21.1	39.3	49.4	28.2	32.5
None	19.7	4.8	8.3	40.9	54.8	64.5
Don’t know	0.7	0.7	0.5	1.0	3.1	0.7
Experiences of friends, relatives (1)						
Satisfactory	55.1	60.2	59	50.7	70.6	71.1
Unsatisfactory	24.3	14.8	19.2	30.1	15.2	20.5
Mixed/Don’t know	20.6	25	21.9	19.2	14.3	8.3
Husband’s approval						
Approve	63.6	77.4	62.6	30.6	29.4	14.0
Disapprove	35.9	22.1	36.5	68.0	70.0	84.9
Don’t know	0.5	0.5	0.8	1.4	0.6	1.1
Mean net positive score (2)	20.3	21.6	19.2	0.9	31.2	5.5
Mean % of don’t know (3)	0.5	0.3	0.5	1.9	1.4	2.3
TOTAL (N)	2787	2808	2789	2664	2792	2423

(1) Amongst those knowing some friends were using the methods

(2) Mean percent difference between percentages of women who gave positive and negative responses among the above attributes except husband’s approval

(3) Mean percent of women who said 'don’t know' to the above attributes except experiences of a method in social network and husband’s approval

**Table 5:** Percentage of women with specific opinions by use status (Nairobi)

Attributes	Pills			Injectables			Implants		
	Current users	Past users	Never users	Current users	Past users	Never users	Current users	Past users	Never users
Access									
Easy	98.3	94.0	90.6	99.1	96.9	93.0	96.7	92.4	87.1
Hard	1.7	6.0	8.8	0.9	3.0	6.3	3.3	7.6	12.5
Don’t know/unsure	0.0	0.0	0.6	0.0	0.1	0.7	0.0	0.0	0.4
Effectiveness									
Yes	93.6	70.9	65.9	97.7	86.0	83.4	98.2	89.0	86.6
No	6.4	29.0	33.7	2.3	14.0	16.2	1.8	11.0	12.9
Don’t know	0.0	0.1	0.4	0.0	0.0	0.4	0.0	0.0	0.4
Cause health problems									
Yes, serious	5.1	14.2	12.6	10.6	23.0	21.1	9.7	35.5	24.5
Yes, not serious	18.4	32.7	28.2	33.4	38.5	39.4	31.9	35.9	37.6
No	76.5	52.9	58.4	55.9	38.5	38.9	58.5	28.4	37.2
Don’t know	0.0	0.2	0.8	0.1	0.0	0.6	0.0	0.2	0.7
Interfere with menstruation									
Yes	25.2	40.7	42.5	73.0	82.3	77.1	59.6	75.1	66.7
No	74.8	59.0	55.9	27.0	17.7	21.0	40.4	24.9	31.9
Don’t know	0.0	0.2	1.6	0.0	0.0	1.9	0.0	0.0	1.4
Cause unpleasant side effect									
Yes	30.3	51.4	47.3	50.6	65.8	61.7	44.8	70.9	60.1
No	69.7	48.6	51.9	49.4	34.2	37.9	55.2	28.9	39.5
Don’t know	0.0	0.0	0.8	0.0	0.0	0.4	0.0	0.2	0.4
Unsafe to use for a long time									
Yes, should take a break	46.2	69.2	74.0	49.0	70.0	69.0	37.9	61.6	61.6
No, safe for long use	53.8	30.8	25.4	50.9	29.9	29.8	61.9	38.4	37.9
Don’t know	0.0	0.0	0.7	0.1	0.2	1.2	0.2	0.0	0.5
Cause infertility									
Yes, perhaps	9.8	13.1	14.7	26.1	30.0	26.9	16.0	22.7	23.4
No	89.3	86.8	84.6	73.6	69.8	72.1	83.4	77.0	75.7
Don’t know	0.9	0.1	0.7	0.3	0.2	1.0	0.5	0.2	0.9
FP use among friends, relatives									
Most	34.2	26.1	15.3	72.6	63.0	52.1	56.3	46.9	30.8
About half	10.7	7.5	6.5	9.2	10.0	10.7	14.9	14.7	13.1
Few	45.3	50.6	54.0	15.2	22.3	26.9	25.9	33.0	44.7
None	9.8	15.2	23.5	2.5	4.2	9.0	2.7	4.6	10.8
Don’t know	0.0	0.6	0.8	0.4	0.5	1.3	0.2	0.7	0.6
Experiences of friends, relatives (1)									
Satisfactory	83.4	52	52.2	74.9	52.9	52.7	75.2	48.8	56
Unsatisfactory	5.2	27.9	25.3	4.6	19.3	20.9	6.6	25.1	21.9
Mixed/Don’t know	11.4	20.1	22.4	20.4	27.8	26.4	18.2	26.1	22.1
Husband’s approval									
Approve	92.3	71.8	55.3	91.5	77.5	58.4	90.0	68.5	53.1
Disapprove	7.69	28.19	43.82	8.4	22.4	39.8	9.8	31.5	45.7
Don’t know	0.0	0.0	0.8	0.1	0.1	1.8	0.2	0.0	1.2
Satisfied with use									
Satisfied	90.2	41.3	–	87.7	44.9	–	86.5	32.3	–
Unsatisfied	8.5	55.8	–	10.5	53.2	–	10.7	64.8	–
Mixed/Neither	1.3	2.3	–	1.3	1.3	–	2.0	2.0	–
Missing	0.0	0.6	–	0.6	0.6	–	0.7	1.0	–
Mean net positive score (2)	53.4	19.8	15.8	38.0	14.1	13.0	44.5	11.8	13.2
Mean % of don’t know (3)	0.1	0.2	0.8	0.1	0.1	1.0	0.1	0.2	0.7
TOTAL(N)	234	869	1684	909	1218	681	549	409	1831

(1) Amongst those knowing some friends were using the methods

(2) Mean percent difference between percentages of women who gave positive and negative responses among the above attributes except husband’s approval

(3) Mean percent of women who said "don’t know" to the above attributes except experiences of a method in social network and husband’s approval

Note: Chi-square test was conducted to assess associations between each of the above attributes and use status. The p-values were all 0.01 or smaller except the perceptions on risk of infertility of pills (*p* = 0.061) and injectables (*p* = 0.015)

**Table 6:** Percentage of women with specific opinions, amongst those who have heard of methods (Homa Bay)

Attributes	Pills	Injectables	Implants	IUD	Condoms (4)	Female sterilisation (5)
Access						
Easy	79.4	90.4	84.5	52.8	93.9	41.7
Hard	12.5	7.9	10.5	25.8	3.6	40.3
Don’t know/unsure	8.1	1.6	5.0	21.4	2.5	18.0
Effectiveness						
Yes	62.6	91.6	90.3	66.1	77.5	82.5
No	23.8	5.6	3.7	8.6	17.4	5.4
Don’t know	13.5	2.8	6.0	25.3	5.1	12.1
Cause health problems						
Yes, serious	37.8	31.2	28.3	36.0	11.4	32.7
Yes, not serious	21.5	31.0	25.9	16.0	11.3	12.2
No	23.0	30.1	30.5	18.7	70.0	31.5
Don’t know	17.7	7.7	15.4	29.3	7.3	23.6
Interfere with menstruation						
Yes	56.5	80.2	60.1	35.6	–	–
No	22.1	14.7	22.5	25.1	–	–
Don’t know	21.4	5.2	17.4	39.2	–	–
Cause unpleasant side effect						
Yes	57.5	63.2	51.7	44.7	14.0	38.3
No	21.6	28.7	30.1	20.9	77.6	36.0
Don’t know	20.9	8.2	18.2	34.4	8.4	25.7
Unsafe to use for a long time						
Yes, should take a break	66.9	68.5	60.8	62.5	37.3	–
No, safe for long time	20.3	26.3	31.7	17.4	56.4	–
Don’t know	12.8	5.2	7.6	20.0	6.3	–
Cause infertility						
Yes, perhaps	15.6	17.1	12.7	16.2	–	–
No	72.9	77.3	78.1	63.4	–	–
Don’t know	11.6	5.7	9.2	20.5	–	–
FP use among friends, relatives						
Most	13.9	64.6	49.1	3.9	30.3	3.3
About half	7.4	8.3	8.7	3.5	7.7	2.7
Few	49.3	19.0	31.8	42.4	26.0	51.3
None	13.8	2.1	3.7	26.9	6.1	26.0
Don’t know	15.7	6.0	6.8	23.4	30.0	16.7
Experiences of friends, relatives (1)						
Satisfactory	38.0	54.1	57.7	39.4	72.9	59.1
Unsatisfactory	28.5	11.2	13.1	27.2	10.6	17.3
Mixed/Don’t know	33.5	34.7	29.2	33.4	16.5	17.1
Husband disapprove						
Approve	45.5	64.4	52.2	25.2	53.3	18.1
Disapprove	43.7	30.2	38.7	58.8	41.2	70.5
Don’t know	10.8	5.5	9.1	15.9	5.5	11.4
Mean net positive score (2)	−2.5	16.6	20.1	−3.4	49.8	5.6
Mean % of don’t know (3)	15.2	5.3	10.7	26.7	9.9	19.2
TOTAL(N)	2358	2405	2391	1973	1338	2080

(1) Amongst those knowing some friends were using the methods

(2) Mean percent difference between percentages of women who gave positive and negative responses among the above attributes except husband’s approval

(3) Mean percent of women who said “don’t know” to the above attributes except experiences of a method in social network and husband’s approval

(4) 1008 responses missing owing to error in electronic data capture program

(5) The question on interference with menstruation was not asked for female sterilisation in Homa-Bay

**Table 7:** Percentage of women with specific opinions by use status (Homa Bay)

Attributes	Pills			Injectables			Implants		
	Current users	Past users	Never users	Current users	Past users	Never users	Current users	Past users	Never users
Access									
Easy	92.3	84.7	77.1	93.7	90.6	87.0	93.9	88.7	81.1
Hard	7.7	12.5	12.7	6.2	8.7	8.4	6.2	8.3	12.1
Don’t know/unsure	0.0	2.8	10.3	0.2	0.7	4.6	0.0	3.0	6.9
Effectiveness									
Yes	86.2	66.8	60.3	97.1	92.1	85.2	98.6	89.9	88.1
No	10.8	28.5	22.8	2.1	7.0	6.8	1.1	5.7	4.0
Don’t know	3.1	4.7	16.9	0.8	0.8	8.0	0.2	4.5	7.9
Cause health problems									
Yes, serious	13.9	42.7	37.0	24.2	36.8	29.4	16.4	36.3	29.9
Yes, not serious	33.9	22.9	20.6	30.1	32.3	30.0	24.6	26.2	26.1
No	46.2	26.2	21.0	40.9	27.7	23.3	51.5	31.9	24.4
Don’t know	6.2	8.2	21.4	4.8	3.3	17.3	7.5	5.7	19.6
Interfere with menstruation									
Yes	40.0	59.6	56.1	80.2	84.1	74.0	62.9	67.9	57.6
No	53.9	32.5	17.4	19.0	14.2	11.2	35.3	24.4	18.7
Don’t know	6.2	8.0	26.5	0.8	1.8	14.8	1.8	7.7	23.7
Cause unpleasant side effect									
Yes	33.9	63.0	56.5	55.3	68.7	62.2	41.0	59.8	53.0
No	60.0	29.0	17.7	39.9	27.8	19.2	51.7	31.9	23.9
Don’t know	6.2	8.0	25.8	4.8	3.6	18.6	7.3	8.3	23.1
Unsafe to use for a long time									
Yes, should take a break	47.7	70.5	66.4	58.0	73.7	70.6	50.8	65.2	62.6
No, safe for long time	40.0	22.4	18.9	37.3	23.6	19.9	47.2	29.8	27.9
Don’t Know	12.3	7.1	14.7	4.7	2.7	9.5	2.1	5.1	9.6
Cause infertility									
Yes, perhaps	15.4	15.6	15.6	15.4	16.8	19.3	10.7	13.4	13.1
No	80.0	77.4	71.1	80.5	80.0	69.8	83.4	81.0	76.1
Don’t know	4.6	6.9	13.4	4.1	3.2	11.0	5.9	5.7	10.8
FP use among friends, relatives									
Most	18.5	19.1	11.9	73.6	66.7	52.6	70.8	53.9	42.1
About half	10.8	8.0	7.1	6.8	7.7	10.6	6.6	6.6	9.7
Few	50.8	45.1	50.6	14.7	17.6	25.3	18.2	30.4	35.7
None	7.7	13.4	14.2	0.9	2.4	2.8	1.8	2.7	4.5
Don’t know	12.3	14.4	16.2	4.1	5.5	8.7	2.5	6.6	8.0
Experiences of friends, relatives (1)									
Satisfactory	65.4	39.7	36.2	69.0	48.2	47.9	77.6	49.2	53.6
Unsatisfactory	11.5	34.9	27.0	4.0	15.4	11.7	3.8	21.6	14.1
Mixed/Don’t know	23.1	25.5	36.7	27.0	36.4	40.4	18.6	29.2	32.3
Husband’s approval									
Approve	76.9	55.6	40.9	77.2	67.5	47.0	77.7	59.8	43.7
Disapprove	20.0	38.4	46.4	20.7	29.1	41.1	20.7	36.0	44.2
Don’t know	3.1	6.1	12.6	2.1	3.4	12.0	1.6	4.2	12.1
Satisfied with use									
Satisfied	76.9	38.4	–	86.1	44.9	–	87.7	31.0	–
Unsatisfied	16.9	43.6	–	6.3	38.7	–	7.1	30.1	–
Mixed/Neither	3.1	6.3	–	5.1	9.0	–	5.2	3.3	–
Missing (4)	3.1	11.8	–	2.4	7.3	–	0.0	35.7	–
Mean net positive score (2)	31.1	−0.3	−4.5	29.7	12.8	9.6	42.1	16.6	14.8
Mean % of don’t know (3)	6.3	7.5	18.1	3.0	2.7	11.5	3.4	5.8	13.7
TOTAL(N)	65	576	1717	662	1066	677	439	336	1616

(1) Amongst those knowing some friends were using the methods

(2) Mean percent difference between percentages of women who gave positive and negative responses among the above attributes except husband’s approval

(3) Mean percent of women who said “don’t know” to the above attributes except experiences of a method in social network and husband’s approval

(4) The missing occurred due to initial technical errors in the electronic data capture

Note: Chi-square test was conducted to assess associations between each of the above attributes and use status. The p-values were all 0.002 or smaller

In Matlab, implants, IUDs and female sterilisation were rarely used and unfamiliarity was reflected in the high proportions of respondents who said “don’t know” on attributes of these methods. Thus over half of women did not know whether these methods might cause health problems, unpleasant side effects or menstrual disruption. However, nearly all women acknowledged these three methods to be effective and two-thirds or more thought access would be easy. Among those knowing social network members who had used these methods, over three-quarters deemed their experience to be satisfactory. Yet a majority considered that their husband would disapprove of using these methods.

Oral contraceptives followed by injectables dominate the method-mix. Pills attracted more favourable opinions than injectables. For instance, 40% and 38% thought injectables might cause health problems and unpleasant side effects, respectively, compared with 24% and 26% for pills. Nearly one-quarter thought that their husbands would disapprove of their use of injectables compared with 8% for pills. The two methods were rated similarly on other dimensions. Both were regarded as easy to obtain and effective. The experience of their social network with use of either method was judged to be satisfactory by over 90%. Very few thought that either method might cause infertility and almost equal proportions considered long term use to be safe.

We compared the views of current, past and never users of pills, injectables and implants (Table 3). The largest consistent difference between current and past users across all three methods was the view that the method caused unpleasant side effects, though appreciable minorities (from 14% for the pill to 30% for the implant) of current users acknowledged side effects. Notably, 29% of past implant users thought that the method caused serious health problems. Furthermore, past implant users were more likely to rate their experience as unsatisfactory than past injectable or pill users.

### Method-specific beliefs and opinions: Nairobi

As indicated by the mean net positive scores, beliefs about methods were less favourable in Nairobi than in Matlab and the proportions giving “don’t know” responses were much lower. With the exception of sterilisation, large majorities considered methods easy to obtain (Table 4). Injectables and implants were judged to be effective by 90%. However, in a clear departure from objective evidence, this level falls to about 70% for IUDs and sterilisation. The view that a method might cause serious health problems was widespread (with the exception of condoms), rising from 13% for pills to 30% for sterilisation and 35% for IUDs. Similarly, about 60% associated injectables, implants, and IUDs with unpleasant side effects, as did over 40% for pills and sterilisation. Only minorities were of the view that the hormonal methods and IUDs could be safely used for a long time without taking a break. Between 14% (pills) and 28% (injectables) of women thought that a method might cause infertility. Despite these views, between 51% and 71% rated the experience of social network members with use of methods as satisfactory. While most women thought that their partners would approve of their use of pills, injectables and implants, perceived approval dropped to 30% for IUDs and condoms and further to 14% for sterilisation.

Analysis of opinions by use status showed sharp divides between current users and past or never users (Table 5). For instance, 11% of current injectable users (the dominant method in this site) believed that the method caused serious health problems compared with over 20% of past or never users. This difference was more pronounced for implants: 10% for current users versus 36% for past users and 25% for never users. Similarly, about three quarters of current users of injectables, pills and implants reported satisfactory experience of their social network compared with about 50% of past and never users. A clear gradient in perceived partner’s approval is apparent for all three methods, with 90% approval for current users, around 70% for past users and 50–60% for never users. Nevertheless, appreciable proportions of current users held negative views about their method: 30%, 51% and 45% of pill, injectable and implant users, respectively, thought that the method caused unpleasant side effects. Around 40% of current users considered their method to be unsafe for prolonged use. Despite these reservations, close to 90% of current users rated their method as satisfactory compared with 32–44% of past users.

### Method-specific beliefs and opinions: Homa Bay

Condoms received a much higher mean net positive score than other methods and perceived partner’s approval of this method was higher in this site with high adult HIV prevalence than in Matlab or Nairobi. The experience of social network members with use of condoms was more likely to be rated as satisfactory than other methods: 73% for condoms compared with 54–58% for the two dominant methods, injectables and implants (Table 6). Condoms were more likely to be considered as effective than pills, though less likely than injectables and implants.

With the exception of condoms, about 30% thought that other methods caused serious health problems; between 38% (sterilisation) and 63% (injectables) considered that methods caused unpleasant side effects and about two-thirds judged methods to be unsafe for prolonged use without taking a break.

As in Nairobi, current users of pills, injectables and implants were much more likely to express positive views about their method than past or never users, except with regard to fears among a minority of women about infertility. However, the prevalence of adverse views even among current users was striking. Thus, 14%, 24% and 16% of current pill, injectable and implant users, respectively, considered that their method caused serious health problems and between 34% and 55% associated the method with unpleasant side effects. These adverse beliefs did not translate into dissatisfaction with the current method: on the contrary, between 77% and 88% judged their method to be satisfactory while only 31–45% of past users were satisfied with the use.

## Discussion

In view of the principle of cognitive dissonance—the tendency to align beliefs and attitudes to behaviour—it is no surprise that most current users of a method consider it to be satisfactory and express more positive views than past or never users. In apparent contradiction to high overall satisfaction is the finding that appreciable proportions of these current users appear to be concerned about serious health effects and acknowledge that the method causes unpleasant side effects. The view that it is unsafe to use a method for a long time without taking a break is widespread among current users, particularly in the Kenyan sites. Follow-up surveys will establish the impact of these perceptions on discontinuation.

Dissatisfaction among past users, who tend to outnumber current users, may be a powerful influence on the overall climate of opinion; indeed the views of past and never users tend to be similar. The low level of overall satisfaction among past users of injectables, pills and implants in Kenyan sites, associated with health worries and side effects, is a concern, particularly with implant users of whom only about 30% in Nairobi reported satisfaction. A continuation of the steep rise in the use of implants in Kenya, therefore, could be in jeopardy. Similarly in Matlab, the results imply considerable potential resistance to long-acting reversible methods (IUDs and implants), the promotion of which is a national priority.

The study is not without limitations. Information on perceived partner’s approval of specific methods is difficult to interpret. While close to 90% reported partner’s support of family planning in general, perceived approval is low in Matlab for use of implants, IUDs and female sterilisation, and also low in the Kenyan sites for use of IUDs and sterilisation. Whether or not, these results reflect women’s own misgivings about these methods remains unclear.

The value of this detailed information in explaining reproductive and contraceptive behaviour is uncertain and a verdict must await the results of follow-up surveys. Nevertheless, the results are of considerable interest because this study, to our knowledge, is the first to have obtained comparable quantitative information in LMICs on attitudes and beliefs on each of eight major family planning methods. Many of the findings from this study are consistent with earlier studies that have documented fears about side effects and damage to health to be widespread. The result regarding the belief that it is unsafe to use a method for a long term without taking a break is simiar to the common behaviours observed in the Philippines [28]. Other findings are new and have considerable possible implications for behaviour.

Moreover, the ability of this study to contrast the beliefs of current, past and never users of the three more prevalent methods represents a major contribution. In 2015 an expert meeting on misperceptions about contraceptives called for a revitalization of discussion on this issue. Participants identified the need for quantitative data that distinguish concerns stemming from documented side effects from those resulting from rumours or myths and the need for evidence about links between misperceptions and method choice [29]. This paper starts to address these gaps in evidence and further progress is expected when follow-up survey data become available.

## Conclusions

In conclusion, high levels of contraceptive use can clearly co-exist with widespread misgivings about methods, even those that are widely used. Serious concerns about damage to health, long term fertility impairment, and dangers of prolonged use without taking a break were particularly common in the Kenyan sites and these beliefs may explain the high levels of discontinuation observed in Kenya and elsewhere in Africa. This documentation of beliefs provides useful guidance for counselling and informational campaigns. The generally negative views of past users imply that programmes may need not only to improve individual counselling but also strengthen community information campaign to change the overall climate of opinion which may have been influenced by dissatisfaction among past users.

## Additional file

Additional file 1: **Table S1.** Enrollment Overview. **Table S2.** General attitudes by site. **Table S3.** Percentage of respondents stating specific characteristics to be ‘very important’ by site. **Table S4.** Percentage of women with specific opinions (traditional methods) by site. (PDF 916 kb)
