# Supplementary material for: Women’s attitudes and beliefs towards specific contraceptive methods in Bangladesh and Kenya
Source: Reprod Health. 2018 May 8;15:75. doi: 10.1186/s12978-018-0514-7 (PMC5941610; doi:10.1186/s12978-018-0514-7)
Supplement: Supplementary file 1 — Table S1. Enrollment Overview. Table S2. General attitudes by site. Table S3. Percentage of respondents stating specific characteristics to be ‘very important’ by site. Table S4. Percentage of women with specific opinions (traditional methods) by site. (PDF 916 kb) [file 12978_2018_514_MOESM1_ESM.pdf]

Additional files

Table S1: Enrolment overview

Criteria	Matlab	Nairobi	Homa Bay
Women identified in the database/household listing	3109	5905	3118
Ineligible women identified at site	121	589	235
Out migration	188	1870	0
Women not found at home after 3 visits	181	132	198
Temporarily away/household not located/had passed away	7	363	0
Refused	4	136	83
Incapacitated	3	3	10
Other*	0	0	168
Eligible women interviewed	2605	2812	2424

*167 women could not be interviewed due to resistance from the villagers and 1 women was partially interviewed.

Table S2: General attitudes by site

	Matlab (%)	Nairobi (%)	Homa-Bay (%)
Respondent's attitudes to FP			
Oppose	0.4	2.7	5.5
Support	99.2	97.3	93.6
Don't know/unsure	0.4	0.1	0.9
Husband's attitudes to FP			
Oppose	1.0	12.3	24.8
Support	97.8	86.6	68.4
Don't know/unsure	1.2	1.1	6.9
Opposition to FP of friends, relatives or neighbours			
Most	1.1	6.3	14.4
About half	0.5	2.5	5.8
Few	2.0	40.1	59.2
None	95.0	50.1	12.0
Don't know/unsure	1.4	1.0	8.7
FP use of friends, relatives or neighbours			
Most	65.5	85.7	78.3
About half	8.2	4.9	5.6
Few	21.6	6.4	11.3
None	2.2	2.3	0.3
Don't know/unsure	2.6	0.7	4.5
Religion			
Oppose	42.9	19.1	29.9
Support	50.9	75.6	46.8
No religion	1.9	1.3	0.2
Don't know/unsure	4.3	4.0	23.1
TOTAL (N)	2605	2812	2424

Table S3: Percentage of respondents stating specific characteristics to be 'very important' by site

	Matlab (%)	Nairobi (%)	Homa Bay (%)
Effectiveness	96.2	96.9	95.2
No health risk	80.7	94	92.8
No effect on menstruation	71.6	87.4	81.4
No unpleasant side effects	75	91	90.3
Easy to use	83.5	90.1	88.4
Clandestine use	61.4	58.4	55
Easy to obtain	82.8	89.2	88.3
Can be used for long time without re-supply	67.3	64.5	83.3
TOTAL (N)	2605	2,812	2424

Table S4: Percentage of women with specific opinions (traditional methods) by site

Attributes	Matlab		Nairobi		Homa-Bay	
	Withdrawal	Rhythm method	Withdrawal	Rhythm method	Withdrawal	Rhythm method
Access						
Easy	-	-	-	-	-	-
Hard	-	-	-	-	-	-
Don't know/unsure	-	-	-	-	-	-
Effectiveness						
Yes	78.8	73.8	26.8	46.0	37.8	47.4
No	11.0	13.2	72.7	53.8	50.1	44.2
Don't know	10.2	13.0	0.5	0.2	12.1	8.4
Cause health problems						
Yes, serious	0.4	1.2	1.0	0.6	3.2	2.9
Yes, not serious	1.6	1.3	6.8	2.6	3.7	2.6
No	59.8	60.6	91.7	96.8	83.2	88.0
Don't know	38.2	36.9	0.5	0.1	10.0	6.5
Interfere with menstruation						
Yes	-	-	-	-	-	-
No	-	-	-	-	-	-
Don't know	-	-	-	-	-	-
Cause unpleasant side effect						
Yes	1.1	2.5	10.0	9.4	5.3	3.8
No	58.6	58.6	89.9	90.5	84.4	89.2
Don't know	40.3	38.9	0.1	0.1	10.4	7.0
Unsafe to use for a long time						
Yes, should take a break	21.4	17.9	59.0	37.0	44.2	35.9
No, safe for long time	48.4	52.2	36.0	61.9	44.6	57.7
Don't know	30.2	29.9	5.0	1.1	11.3	6.4
Cause infertility						
Yes, perhaps	-	-	-	-	-	-
No	-	-	-	-	-	-
Don't know	-	-	-	-	-	-
FP use among friend, relatives						
Most	1.3	0.3	1.7	4.1	1.9	6.0
About half	0.0	0.0	0.3	2.3	2.4	4.8
Few	11.8	11.3	19.4	50.3	25.8	36.4
None	7.9	8.9	73.2	42.1	21.3	21.3
Don't know	79.0	79.5	5.3	1.1	48.6	31.5
Experiences of friend, relatives						
Satisfactory	9.0	8.5	10.7	33.6	10.0	23.5
Unsatisfactory	0.6	0.4	7.8	11.8	12.3	12.0
Mixed	0.8	0.6	2.7	11.4	6.4	10.7
No users known	7.9	8.8	73.2	42.1	21.3	21.3
Don't know	81.7	81.6	5.5	1.1	50.1	32.5
Husband's approval						
Approve	38.2	46.8	17.1	55.0	27.6	45.0
Disapprove	46.4	34.6	76.9	38.8	52.6	37.2
Disapprove all methods	0.2	0.4	5.6	5.7	8.0	7.9
Don't know	15.2	18.2	0.5	0.5	11.7	9.9
TOTAL(N)	1423	1853	1,721	2,511	1252	1724

Note: Opinions about access, interference with menstruation, and causing infertility were not asked as they are not relevant for the traditional methods.
